# The Role of Hospital and Community Pharmacists in the Management of COVID-19: Towards an Expanded Definition of the Roles, Responsibilities, and Duties of the Pharmacist

**DOI:** 10.3390/pharmacy8030140

**Published:** 2020-08-07

**Authors:** Nicola Luigi Bragazzi, Muhammad Mansour, Alessandro Bonsignore, Rosagemma Ciliberti

**Affiliations:** 1 Department of Health Sciences (DISSAL), Postgraduate School of Public Health, Genoa University, 16132 Genoa, Italy; 2 Laboratory for Industrial and Applied Mathematics (LIAM), Department of Mathematics and Statistics, York University, Toronto, ON M3J 1P3, Canada; 3 Department of Surgery A, Galilee Medical Center, Nahariya, Faculty of Medicine of the Galilee, Bar-Ilan University, Safed 5290002, Israel; hmode_220@hotmail.com; 4 Division of General Surgery, St. Michael’s Hospital, Unity Health Toronto, University of Toronto, Toronto, ON M5B 1W8, Canada; 5 Section of Legal and Forensic Medicine, Department of Health Sciences (DISSAL), University of Genoa, 16132 Genoa, Italy; alessandro.bonsignore@unige.it; 6 Section of History of Medicine and Bioethics, Department of Health Sciences (DISSAL), University of Genoa, 16132 Genoa, Italy; rosellaciliberti@yahoo.it

**Keywords:** COVID-19, viral outbreak, community and hospital pharmacists, history of pharmacies and pharmacists

## Abstract

Since late December 2019, a novel, emerging coronavirus was identified as the infectious agent responsible for a generally mild but sometimes severe and even life-threatening disease, termed as “coronavirus disease 2019” (COVID-19). The pathogen was initially named as “2019 novel coronavirus” (2019-nCoV) and later renamed as “Severe Acute Respiratory Coronavirus type 2” (SARS-CoV-2). COVID-19 quickly spread from the first epicenter, the city of Wuhan, province of Hubei, mainland China, into neighboring countries, and became a global pandemic. As of July 15th 2020, the outbreak is still ongoing, with SARS-CoV-2 affecting 213 countries and territories. The coronavirus has caused a dramatic toll of deaths and imposed a severe burden, both from a societal and economic point of view. COVID-19 has challenged health systems, straining and overwhelming healthcare facilities and settings, including hospital and community pharmacies. On the other hand, COVID-19 has propelled several changes. During the last decades, pharmacy has shifted from being products-based and patient-facing to being services-based and patient-centered. Pharmacies have transitioned from being compounding centers devoted to the manipulation of *materia medica* to pharmaceutical centers, clinical pharmacies and fully integrated “medical-pharmaceutical networks”, providing a significant range of non-prescribing services. Moreover, roles, duties and responsibilities of pharmacists have paralleled such historical changes and have known a gradual expansion, incorporating new skills and reflecting new societal demands and challenges. The COVID-19 outbreak has unearthed new opportunities for pharmacists: community and hospital pharmacists have, indeed, played a key role during the COVID-19 pandemic, suggesting that a fully integrated, inter-sectoral and inter-professional collaboration is necessary to face crises and public health emergencies. Preliminary, emerging evidence seems to suggest that, probably, a new era in the history of pharmacies (“the post-COVID-19 post-pharmaceutical care era”) has begun, with community pharmacists acquiring more professional standing, being authentic heroes and frontline health workers.

## 1. The Ongoing COVID-19 Outbreak

Since late December 2019, a novel, emerging coronavirus was identified as the infectious agent responsible for a generally mild but sometimes severe and even life-threatening disease, termed as “coronavirus disease 2019” (COVID-19). The pathogen was initially named as “2019 novel coronavirus” (2019-nCoV) and later renamed as “Severe Acute Respiratory Coronavirus type 2” (SARS-CoV-2). COVID-19 quickly spread from the first epicenter, the city of Wuhan, province of Hubei, mainland China, into neighboring countries, and became a global pandemic [1].

As of July 15th 2020, the outbreak is still ongoing, with SARS-CoV-2 affecting 213 countries and territories. The coronavirus has caused a dramatic toll of deaths (approximately 580,000 deaths) and imposed a severe burden, both from a clinical, societal and economic point of view, with more than 13 million cases.

Given that as of today there are no effective vaccine products and drugs to prevent and treat the COVID-19 infection, respectively, countries have implemented behavioral, non-pharmacological interventions (NPIs), including the closure of non-essential businesses, as well as of schools and universities, travel restrictions, such as the cancellation of domestic and international flights, self-isolation, social and physical distancing, quarantine, and even lockdown of entire communities and territories. All these measures have been enforced in the effort to curb the outbreak [2].

COVID-19 has significantly challenged the health sector, and health systems in many states around the world have revealed their weaknesses. 

Globally, they have been overwhelmed and strained, experiencing unprecedented pressure and scarcity of human resources (nurses and physicians, as well as other allied health workers, including hospital and community pharmacists), and equipment (respirators and ventilators, alcohol-based hand sanitizers, and personal protective equipment or PPE, like face and surgical masks and gloves, among others). 

Our constitutive condition of vulnerability has suddenly and dramatically become the center of collective attention. Death, usually expelled from the everyday life of contemporary society, has brutally highlighted our fragility and how our control of external situations is weak.

In this context of emergency full of uncertainties, new complex questions have suddenly hit the community urging the assumption of ethical responsibility on multiple issues: (i) whether to implement a package of stringent public health measures, such as NPIs (in particular, self-isolation, social and physical distancing, quarantine and lockdown of entire communities and territories) as an effective health strategy to curb the spread of the outbreak; (ii) to control the mobility of people and to enforce limitation measures that impact on personal freedoms; (iii) the need to collect and use citizens’ personal data; (iv) the imbalance between needs and available resources; (v) the identification of the selection criteria for the distribution of PPE; (vi) the definition of the categories that should be primarily subjected to diagnostic tests; (vii) the allocation of beds of intensive care; (viii) the election of patients that should undergo mechanical ventilation (invasive and non-invasive); and (ix) the choices related to the use of experimental or off label therapies, among others.

Despite the non-pharmacological nature of these public health policies, hospital and community pharmacists have played and are still playing a key role in the containment and management of the COVID-19 crisis, which has represented a real, unique challenge, from an economic, social, cultural, political, health and ethical perspective.

The next paragraphs will briefly overview the history and the evolution of the roles, responsibilities and duties of the pharmacist and the contributions of the community and hospital pharmacies during the COVID-19 pandemic.

## 2. The Pharmacist: Past, Present and Future

In the last decades, the professional figure of the pharmacist has known a gradual expansion of their roles, duties and responsibilities. If, initially, the role was essentially “products-based”, “patient-facing” and mainly consisted in dispensing prescribed therapies, in the last decades it has shifted towards being “services-based”, “patient-centered”. The same type of services offered by the pharmacists is gradually expanding towards not strictly pharmaceutical areas, with the delivering of a range of non-pharmacological services, such as patient advice and counseling [3].

**Figure 1:**
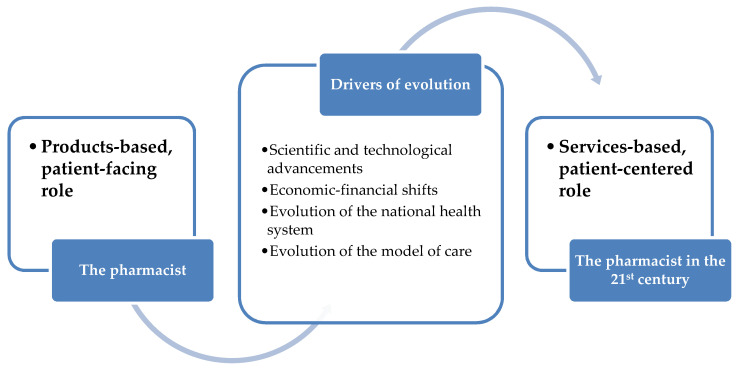
The historical evolution of the roles, responsibilities and duties of the pharmacist.

Several drivers of evolution have facilitated and contributed to this transition: (i) scientific advancements and technological achievements; (ii) economic and financial shifts with the necessity of implementing new business models; (iii) evolution of the healthcare settings and of the national health systems; and (iv) evolution of the model of care, which is currently more focused on the patients and their needs (the so-called “proactive, tailored, personalized or individualized medicine” instead of a “one-size-fits-it-all and reactive” framework) (Table 1 and Figure 1) [4,5].

Ambitious programs, like the “Pharmacists as Personalized Medicine Experts” (PRIME) project led by doctors Lisa McCarthy and Beth Sproule in Canada, have been training the next generation of pharmacists towards evidence-, informed-based, optimized medication therapy prescription, exposing them to advanced and sophisticated concepts, such as pharmacogenetics and pharmacogenomics.

Nowadays, the pharmacist is not considered anymore as a mere expert in medicines and drugs, but represents a relevant healthcare-related actor who plays, in collaboration with other healthcare professionals, a significant role in divulging high-quality information yet intelligible by the laypeople, as well as in monitoring and surveillance of adverse reactions of drugs and medicines (the so-called pharmacovigilance, phytovigilance, and vaccinovigilance).

As such, the pharmacist serves as “privileged information hub”, increasing the health literacy of the community and educating, counseling and empowering citizens, especially those vulnerable and at-risk groups. Therefore, the pharmacist guarantees the correct use and the proper storage and dispensing of the prescribed medicines and promotes the patient’s adherence to medical therapies [6,7,8,9,10].

In several western countries, the pharmacist workforce is actively engaged in enhancing community health and wellbeing: This strong commitment translates in an inter-professional and inter-sectoral collaboration of pharmacists from both public and private pharmacies, operating in agreement with the national health system, with general practitioners, free-choice pediatricians, family physicians and other relevant specialists (Table 1).

Urick and Meggs [9], with a particular focus on the history of the American community pharmacy in the modern era, have identified four main periods: (i) from 1920 to 1949 (termed as the “Soda Fountain Era”); (ii) 1950–1979 (named as the “Lick, Stick, Pour and More Era”); (iii) 1980–2009 (the “Pharmaceutical Care Era”); and (iv), from 2010 onwards (the current era, also known as the “Post-Pharmaceutical Care Era”). The first era was characterized by the great popularity of soda fountains, with pharmacists being mainly engaged in activities, such as compounding and prescription dispensing. The second period was, instead, characterized by the transition from compounding to the dispensing of pre-manufactured proprietary drugs and medicines, with the introductions of new therapeutics just being discovered. Pharmacists began to acquire a more active role in patients counseling, thanks also to pioneering figures like Eugene V. White, the revolutionary father of clinical pharmacies. These new roles, responsibilities and skills are even more acknowledged during the third period, in which the pharmacist is seen as a healthcare professional, whose duties are to take care of patients, trying to improve their perceived quality of life, reduce or possibly eliminate their symptoms, and to manage or prevent disease. Finally, in the currently ongoing period, the community pharmacist is responsible for the provision of “pharmaceutical care services”, also known as “medication therapy management services”, including vaccination campaigns, health screenings, and programs aimed at targeting unhealthy lifestyles and promoting behavioral changes, like smoking cessation.

**Figure 2:**
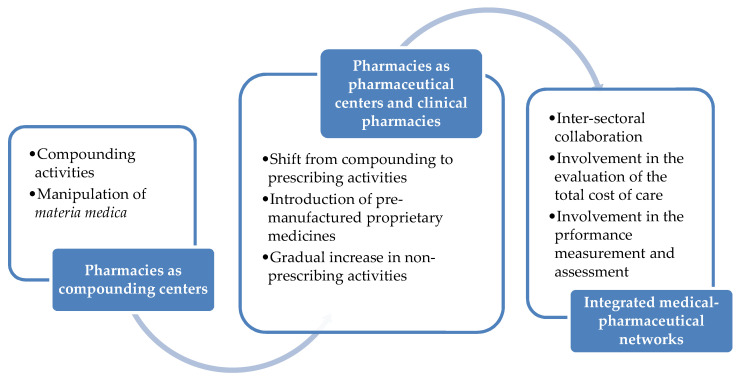
The historical evolution of the pharmacy.

It can be anticipated that in the near future, more non-dispensing services will be assigned to community pharmacists, who will play an increasing role in collaborating with health economists and payers to assess medical expenditures and evaluate the total cost of care, as well as with epidemiologists, biostatisticians, health decision- and policy-makers to quantitatively measure health-related performances. With pharmacists being directly involved in the processes of ensuring cost-effective services while preserving their quality, the transition of pharmacies as “compounding centers” to “pharmaceutical centers”, “clinical pharmacies” and “integrated clinical-pharmaceutical networks” (as pictorially shown in Figure 2) will be completed and will become a daily reality.

Nowadays, community pharmacists are being recognized as health professionals, as well as patient educators and counselors, mentors and researchers/scholars (thanks also to the significant changes in their university education and academic programs), managers and leaders, developers of new, creative business formulas, and healthcare-related actors and stakeholders (Table 1).

From an ethical and professional point of view, inter-professional and inter-sectoral collaboration in promoting health and prevention is a key factor in reducing inequalities and achieving the highest level of health [11]. This collaboration is extremely crucial and vital, especially during crises, such as the ongoing COVID-19 pandemic, in which ethical and societal issues (i.e., inequity in access to healthcare services and related distortions) are even amplified [12].

Improving the outcome of therapeutic strategies, increasing patient health education, optimizing the use of resources and countering the improper use of emergency services, therefore, constitute an essential ethical imperative. Klepser et al. [13] have shown the value of collaborative agreements between the physician and the community pharmacist for treating influenza-like illness (ILI) timely, to reduce the health expenditure arising by patients seeking care in an emergency department for causes that would require only symptomatic management. Similarly, Chin et al. [14] have described the role of Canadian pharmacists in drug distribution, drug information and supporting direct patient care in two outbreaks of the “Severe Acute Respiratory Syndrome” (SARS).

As we will see, preliminary, emerging evidence seems to suggest that, probably, after the four major periods that have characterized contemporary pharmaceutical practices (the “Soda Fountain Era”, the “Lick, Stick, Pour and More Era”, the “Pharmaceutical Care Era”, and the “Post-Pharmaceutical Care Era”), a new era in the history of pharmacies that we could term as “the post-COVID-19 post-pharmaceutical care era” has begun, with community pharmacists acquiring more professional standing, being authentic heroes and frontline health workers.

However, there still exist legal barriers and regulatory obstacles to the full implementation and expansion of pharmacists’ rules, duties, and responsibilities [15,16]. Regarding a potential additional overview of regulatory challenges, it should be emphasized that, during the viral outbreak, pharmacists have been allowed by executive orders to expand their scope of practice (SOP). As such, the main question is: once these executive orders/COVID-19 die down, what will remain of this increased scope?

All these aspects will be overviewed in more detail in the next paragraphs.

## 3. The Role of Hospital and Community Pharmacists during the COVID-19 Crisis: A Prompt, Quick Response

Hospital and community pharmacist communities have quickly reacted to the ongoing pandemic [17,18]. On February 5th 2020, the “International Pharmaceutical Federation” (FIP) has published a guidance entitled “Coronavirus 2019-nCoV outbreak: Information and interim guidelines for pharmacists and the pharmacy workforce”.

These guidelines have been adapted based on the context and setting of each country, for instance, the Chinese Pharmaceutical Association (CPA) has issued a separate expert consensus, entitled the “Coronavirus SARS-CoV-2 Infection: Expert Consensus on Hospital Pharmaceutical Work Guidance and Prevention and Control Strategies”, followed by the “Coronavirus SARS-CoV-2 Infection: Expert Consensus on Work. Guidance and Prevention and Control Strategies for Retail Pharmacies” [17].

In the United States, the American Medical Association (AMA), the American Pharmacists Association (APA), and the American Society of Health-System Pharmacists (ASHSP) have issued similar guidelines [17].

## 4. The Role of Hospital and Community Pharmacists during the COVID-19 Crisis: Ensuring the Drug Supply Chain

During the outbreak, pharmacists have continued ensuring a stable supply of drugs and medicines, establishing close contacts with pharmaceutical companies and manufacturers when necessary, and providing new medicine refill services, such as home delivery of pharmaceuticals for the elderly, immunosuppressed patients or those suffering from chronic-degenerative disorders, or direct supply via community pharmacies instead of accessing outpatient or hospital pharmacies. Besides refill extensions, therapeutic substitution has been another service provided by pharmacists during the outbreak. Guaranteeing continuity of care is, indeed, extremely important during crises, especially in rural and underserved areas [17].

The COVID-19 pandemic has implied profoundly modifying, adapting or even repurposing typical pharmaceutical models, and inventing new approaches. McConachie et al. [18] conducted an observational study of operational and clinical inpatient pharmacy services at a community teaching hospital in the United States of America. Authors found that the number of new order verifications decreased by 30%, namely, from 5000 to 3300 orders per day (*p* < 0.01). Average daily pharmacokinetic dosing consults decreased as well (*p* < 0.01). On the other hand, the overall number of daily pharmacist interventions and services remained stable, since the reduced orders were compensated by an increase in dispensing of drugs, such as anti-malarials (hydroxychloroquine), anti-coagulants (enoxaparin), antibiotics (azithromycin), and sedative drugs (*p* < 0.01).

Pharmacists have also made their best efforts to guarantee a stable supply of alcohol-based hand sanitizers and PPE, such as gloves and masks.

Altogether, these new roles lead to the establishment of what the Chinese health workers and officials have defined as a “pharmacy emergency support guarantee system”, which systematically aims to implement mechanisms and strategies to cope with drug shortages through active surveillance, early alerts and warnings, ensuring drug emergency supply and distribution, monitoring the safety profile of prescribed medicines, reporting eventual side-effects and delivering an event- and data-driven pharmaceutical care during the COVID-19 pandemic [19].

For this aim, pharmacists and healthcare providers can rely on digital tools, based on Big Data and Artificial Intelligence [20]. Big Data is characterized by the *3* Vs: (i) volume, in that they represent a massive and unprecedented wealth of data, exceeding conventional storage and processing capacity; (ii) velocity, in the sense that Big Data enables a fast and real-time monitoring (“Fast Data”); and (iii) variety, which is a quality referring to the several types of data categories and the various channels and sources that can generate, produce and release them.

A recent study by Crowson and colleagues [21] has demonstrated a statistically significant correlation between the volume of National United States Medicaid prescriptions and related web searches, as captured by means of Google Trends (GT, an open-source instrument used for real-time tracking and monitoring of digital activities and behaviors). As such, Big Data Analytics (BDA) enables to forecast and now-cast healthcare utilization trends of a given drug and can be employed for administrative and organizational-logistic purposes (i.e., stockpiling).

The feasibility of exploiting Big Data and AI has also been shown by other studies, which have investigated the digital interest for various drugs [22,23,24], including statins [23]. In other words, the web search query volume can be considered as a proxy of pharmaceutical utilization, changes in prescribing patterns and potential side-effects [22,23,24].

Therefore, pharmacists could monitor digital activities related to COVID-19 medications, like chloroquine, hydroxychloroquine, antibiotics, anti-virals and anti-retrovirals. For instance, Springer and colleagues [25] documented a medium Pearson correlation coefficient (r = 0.300, *p* < 0.05) between “remdesivir” and COVID-19.

Moreover, to the previously mentioned *3* Vs (volume, variety, and velocity) further Vs have been added—including variability, veracity, and value. Indeed, not all available information concerning COVID-19 is reliable and accurate, but, being misleading, could be potentially harmful. Several fake news and post-truth statements can be consumed by searching the Web: These allegations and scans known as “*infodemic*” risk jeopardizing the efforts of the entire community to fight the pandemic [26]. Kouzy and colleagues [27] have analyzed a body of 673 tweets related to COVID-19, generally posted by informal individuals or groups. The majority of them included content that was classified as serious and genuine. Similar results could be found in another study by Rovetta and Bhagavathula [28], confirming the dangers of the circulation and propagation of fake news and their profoundly dramatic impact on public health communication.

Being patients counselors and educators [29], pharmacists have the onus to counteract such a flood of misinformation and rumors related to COVID-19: they could ask their clients about their digital health-seeking behaviors (if they search the web, what and where they search) and refer them to official public health authorities and organisms websites [30,31,32].

Gunther Eysenbach [33], the father and pioneer of infodemiology and infoveillance, has identified four pillars for managing *infodemic*: (i) a constant, real-time information monitoring, able to capture high-quality information and separate it from outright misinformation; (ii) developing and enhancing eHealth knowledge and literacy, as well as science literacy capacity; (iii) promoting and facilitating informed-based processes, like peer-review and fact-checking; and (iv) providing reliable, updated knowledge synthesis and translation, possibly biases- and conflict of interests-free. Recently, a group of six students, including Sameera Toenjes, Avery Loi, Truong Dao, Christopher Tse, Joseph Li and Arvind Grewal, enrolled at the fourth year of the PharmD program at the University of Toronto, Toronto (Canada), developed an evidence-based medicine (EBM)-based initiative termed as “COVID-19 Drug Evidence Initiative” (CDEI), aimed at identifying, appraising and divulging in an accessible way the latest available evidence concerning clinical trials against COVID-19.

## 5. The Role of Hospital and Community Pharmacists during the COVID-19 Crisis: Tele-Pharmacy and Tele-Health Consulting

From a technological point of view, in the healthcare sector, as well as in other sectors, COVID-19 has highlighted the potential role of digital medicine and the use that can be made of it to improve healthcare, cut distances and bring closer people, minimizing physical contact and easing some forms of bureaucracy that slowed down the healthcare processes.

Pharmacy services could be enhanced and improved by means of digital technologies, the so-called tele-pharmacy and tele-health consulting [34,35,36,37]. For instance, pharmacists working at the “Tongji Medical College” at the Huazhong University of Science and Technology, mainland China, have launched the “Online Pharmaceutical Monitoring” service, which has represented a pilot, online pharmaceutical service model, exploiting smart mobile-based applications, such as “WeChat App”. Since these pioneering experiments, also other community pharmacists worldwide have begun moving some of their appointments online and seeing some of their clients by means of online video or phone calls. These have also been employed to follow-up patients, ensuring they have properly understood prescriptions and are compliant with the pharmacological treatment [34].

In Charlotte, North Carolina, United States of America, the Specialty Pharmacy Service at Atrium Health established a partnership with the Leukemia and Myeloid Malignancies Division at the Levine Cancer Institute-Morehead to successfully implement a tele-health consult service for patients suffering from myelofibrosis [36]. Similar experimentations have been performed in other countries, such as Spain [37] and European countries. Customers and patients were highly satisfied with these services [34,35,36,37].

Furthermore, Li et al. [34] have highlighted the role of clinical pharmacists to provide medication education and medication therapy management to patients, providing psychological counseling to the public through a variety of methods including tele-health consultation. The active development/launch of innovative remote pharmaceutical services also with respect to home quarantined patients with chronic diseases suggests the importance of professionals consultation during the COVID-19 pandemic.

## 6. The Role of Hospital and Community Pharmacists during the COVID-19 Crisis: Pharmacists as Information Hubs

Pharmacists, moreover, can serve as an information hub, counteracting the proliferation of falsified medicines, and combating the spread of fake news and medication misinformation concerning the COVID-19 pandemic. Together with territorial medicine and hospitals, pharmacies are fundamental points of reference able to assist the health authorities in promoting correct public information and adopting adequate and responsible behavior on how to prevent the spread of the virus.

According to a cross-sectional study carried out by Hoti and colleagues [38], slightly less than 90% of community pharmacists have a sufficient and adequate knowledge of COVID-19-related preventative measures and correctly implement them. Most pharmacists utilize mobile devices to access COVID-19 related information and actively counteract the dissemination of misleading information concerning COVID-19. Another questionnaire-based study performed by Hamza and coworkers [39] has replicated such results, finding that senior pharmacy students consult social media followed by published articles and television and have positive attitudes and practices towards the COVID-19 pandemic, believing that the outbreak can be successfully controlled and mitigated. Students, in particular females, thoroughly follow recommendations and avoid attending crowded places, even though approximately 50% of the interviewees do not wear masks when leaving their house and going outside. Finally, also the study by Karasneh and collaborators [40] demonstrated a good basic disease knowledge among pharmacists, with a high risk perception. Besides gender, socio-economic and marital status, watching the media as a preferred source of information contributed to shaping and influencing risk perception.

Social media can deliver information, but also contribute to misinformation, becoming a toxic source of fake and post-truth news [41].

In an emergency context full of uncertainties, sudden changes and high emotions, the dissemination of false news on the acquisitions achieved and/or on the drugs concerning the COVID-19 pandemic can seriously damage public health and the relationship of trust with the scientific community. This relationship is a fundamental premise for achieving an adequate level of adherence to the provisions issued by political decision-makers.

In Italy, for example, the National Pharmacy Owners Federation (Federfarma) has disseminated on its social channels communications and posts aimed at highlighting the widespread and reassuring presence of pharmacies throughout the national territory, inviting their sharing in order to amplify the impact of their message and to allow its maximum diffusion. In addition, pharmacies have been involved as the main health unit in an epidemiological project of seroprevalence initiated by the Ministry of Health and the National Institute of Statistics (Istat) to map the country’s epidemiological situation concerning the SARS-CoV-2 virus infection. The sample design involves 2015 municipalities across the national territory and 150 thousand individuals. Through the investigation, detailed information will be obtained to estimate the size and extent of the infection in the population and describe its frequency (incidence and prevalence) in relation to certain factors, such as gender, age, region of belonging, or economic activity. Pharmacies play a key role in the awareness-raising and information campaign of the start of the survey, its purposes and the methods of its implementation, stimulating the participation of the selected subjects.

## 7. The Role of Hospital and Community Pharmacists during the COVID-19 Crisis: Reporting and Referral of COVID-19 Cases

Community pharmacists could also help in reporting and referring suspected COVID-19 cases. However, this practice seems not so common. According to a recent survey conducted by Bahlol and Dewey [42], only 8.8% of interviewees had referred potential infected cases, despite a high level of knowledge and awareness of COVID-19, as also reported by previously mentioned studies.

In some countries or at least in some contexts, community pharmacists have been directly involved in the frontline, being part of the emergency teams, assessing the risk and managing infected cases. In particular, pharmacists have been/are engaged in activities, such as physical assessment, blood pressure monitoring, fever surveillance, and point-of-care testing, among others [43,44]. In North America, for instance, many large chains, such as Rite Aid Corporation or CVS, have been rolling out point-of-care testing at their community pharmacies where patients can drive in and get tested.

## 8. The Role of Hospital and Community Pharmacists in Clinical Experimentations and Trials during the COVID-19 Crisis

The development of the clinical pharmacological experimentation within the international context has also led to an increasingly important role of the professional figure of the pharmacist. Luisetto et al. [45] pointed out that general clinical improvement was achieved thanks to the stable presence of a clinical pharmacist in many medical teams.

The European Good Clinical Practice (GCP) Code establishes (article 4.6.2) that, where allowed/required, the investigator/institution may/should assign some or all of the investigator’s/institution’s duties for investigational product(s) accountability at the trial site(s) to an appropriate pharmacist or another appropriate individual who is under the supervision of the investigator/institution. In addition, article 4.6.3 settles that “The investigator/institution and/or a pharmacist or another appropriate individual, who is designated by the investigator/institution, should maintain records of the product’s delivery to the trial site, the inventory at the site, the use by each subject, and the return to the sponsor or alternative disposition of unused product(s). These records should include dates, quantities, batch/serial numbers, expiration dates (if applicable), and the unique code numbers assigned to the investigational product(s) and trial subjects”.

Within the ethical committees of clinical trials, the pharmacist plays a key role both in the authorization phase of the protocol, and in the subsequent one of the stipulation of the contract between the promoter/sponsor and the Health Authority (e.g., verifying the local feasibility of the experimentation in relation to the available resources and to the structures present, verifying that no additional serious burden is imposed on the company and that the drug is actually provided by the sponsor if provided). In the case of non-profit protocols, during the discussion, the pharmacist is required to verify the existence of the non-profit requirements of the study itself. 

In the Voluntary Harmonization Procedures (VHP) designed for clinical protocols that take place in different European Union states (Regulation 536/2014 concerning clinical trials on medicinal products for human use), the hospital pharmacist also plays an important role of a connection/bridge between the promoters, the competent authority and the local scientific officers with the aim of testing the harmonized evaluation model of clinical trials in the European Union. 

Globally, during the COVID-19 pandemic, pharmacists have played an essential role in facilitating the investigation of novel experimental agents in controlled studies for the prophylaxis and treatment of COVID-19 and in helping obtain medication through compassionate use protocols. In particular, pharmacists have played a key role to support the assessment of patients for eligibility for new agents via compassionate use, in order to acquire the favorable opinion of the Ethics Committee for the use of an emergency investigational new drug, collaborating with local investigational drug services and with the physician or other sponsors to ensure timely delivery of drugs to the patient and eventually speed the time to therapy. 

In addition, as indicated by Gross et al. [46], the critical approach of pharmacists has played an essential role in reviewing and interpreting the information of drugs approved for other indications and repurposing them for COVID-19, to provide clinical and logistical support. The ability of the pharmacist is also to promote drugs with longer half-lives (such as atenolol versus metoprolol and ceftriaxone once a day for the treatment of pneumonia and urosepis) and to evaluate whether to reduce the frequency of clinical staff contacts.

## 9. The Role of Hospital and Community Pharmacists during the COVID-19 Crisis: Addressing Health Inequity

Nearly two decades since the report “Unequal Treatment: Confronting Racial and Ethnic Disparities in Health Care” (2002) that indicated that numerous factors can contribute to health care disparities, providing the adequate recommendations to help remove them, health equity is a goal still to be achieved. In fact, numerous disparities studies showed that racial and ethnic minorities tend to receive a lower level of care and poorer quality of care than non-minorities and that the morbidity and mortality rates from various chronic diseases are greater for the patients of minority ethnicity than non-minorities. 

The “2020 World Health Statistics report” has indicated that, even though overall access to essential health services improved from 2000 to 2017, the service coverage in low- and middle-income countries remains well below the coverage provided in wealthier ones and that only between one third and half of the world’s population was able to obtain essential health services in 2017. In addition to the need to rebuild health systems, the report urges services, such as routine vaccinations and basic hygiene and sanitation. 

The COVID-19 pandemic has shown that ethnic minorities, such as African American and Latino individuals, as well as American Indian, Alaska Native, and Pacific Islander populations, have been hit hard. Disparities and inequities in healthcare generate significant moral and ethical dilemmas, resulting in excess health care expenditures and charging health workers with particular responsibilities.

Moreover, the COVID-19 pandemic is having a serious economic-financial burden, impacting on the allocation of resources that are already limited and constrained. Vulnerable and frail groups are having difficulties in accessing to healthcare provisions, not being able to afford health-related expenses. Furthermore, community and hospital pharmacists can help achieve universal health coverage (UHC), ensuring access to high-quality services, without exposing individuals to financial hardship and out-of-pocket (OOP) expenditure. UHC can be defined as ensuring that all people can benefit from those high-quality health services they need (including prevention, promotion, treatment, rehabilitation, and palliative care). UHC has, therefore, become a major goal for health reforms in many countries and a priority objective of the WHO.

UHC is, indeed, among the “Sustainable Development Goals” (SDGs), aimed at profoundly transforming health systems and making them more resilient, which is of paramount importance, especially during critical situations, such as natural disasters, hazards and other crises, including the ongoing viral outbreak [12].

However, these important goals should be compared with the current data that indicate that over 65% of all countries have less than five pharmacists per 10,000 population.

## 10. The Role of Hospital and Community Pharmacists during the COVID-19 Crisis: Redefining and Inventing Skills and Responsibilities

The expanded roles, responsibilities and duties of hospital and community pharmacists during the COVID-19 crisis are summarized in Table 2.

## 11. Towards an Expanded Definition of the Roles, Responsibilities and Duties of the Pharmacist: Societal Involvement and Engagement

Pharmacists have been directly involved in the frontline, in the establishment of national and international emergency taskforces and public health rapid response teams. Furthermore, during the outbreak, community pharmacists have also provided services usually outside their scope and traditional professional routine. Measures, such as the lockdown and quarantine, have resulted in a dramatic increase in verbal and physical assault.

In countries, like France, Span and the UK, community pharmacists have provided significant psycho-social support to the victims of domestic abuses and violence, acting as port-of-call, and have promptly reported such episodes to the police and relevant authorities. Code-words, such as “mask19”, have been utilized for communicating with the pharmacists over the counter when the victim was accompanied by the spouse.

## 12. Towards an Extended Definition of the Roles, Responsibilities and Duties of the Pharmacist: The Post-COVID-19 World

The still-ongoing COVID-19 pandemic represents an unprecedented event—reminiscent of phenomena, such as the Black Death and the Plague, that we have forgotten about, or have only read about in textbooks. COVID-19 has disrupted a globalized, highly interconnected and urbanized society, modifying our habits and lifestyles.

Community pharmacists are expected to play a key role in the post-COVID19 world, being engaged in re-building public trust and confidence.

Together with other health allied professionals, they will follow the steps of the gradual easing and lifting of the lockdown-induced strictures. These steps will be implemented carefully since the resumption of daily working and social activities are anticipated to result in further outbreak waves/relapses, due to the re-increasing of contact rates and social mixing.

Currently, there are no effective drugs or vaccine products that can be utilized to treat and prevent COVID-19, respectively. Two major trials, including the “Solidarity trial for treatments” (SOLIDARITY), a phase III-IV trial organized by the WHO, are currently ongoing. The “Randomized Evaluation of COVID-19 Therapy” (RECOVERY), based at Oxford, UK, represents one of the biggest randomized controlled trials, systematically assessing a potentially effective drug against COVID-19. Pharmacists are actively involved in clinical trials aimed at discovering effective investigational agents against COVID-19. They can follow the manufacturing, packaging and distribution of medicines, can double-check drug dispensing, take care of the appropriate storage of pharmaceuticals, as well as they can ensure good clinical practices and the observance of regulatory aspects. Pharmacies are also helping in financing studies and researches against COVID-19. For instance, the Spanish Society of Hospital Pharmacy (SEFH) decided to sponsor several clinical trials, with approximately 13% of the Departments of Hospital Pharmacy (DHPs) leading at least one investigation. Furthermore, the Spanish Agency for Medicines and Medical Devices (AEMPS) adopted a fast-track review process for assessing the effectiveness and safety profile of drugs and eventually authorize them. However, a broader involvement of all relevant stakeholders would be of fundamental importance.

Moreover, pharmacists can offer their expertise in medicines and drug-drug interactions. There exist valuable curated databases that enable scholars to explore drug-drug interactions, such as the “Curated Corona Drug Interactions Database for SARS-CoV-2” (CORDITE) developed by Martin and colleagues.

Furthermore, it can be anticipated that pharmacists will play a key role in late Fall/Winter 2020, during the immunization campaigns against influenza. In countries in which COVID-19 induced restrictions have been eased and relaxed too soon, second waves and relapses are more likely to occur. In order to avoid strained and overwhelmed healthcare facilities, vaccinating against influenza will be of paramount importance [47,48,49,50,51,52,53,54].

These initiatives have shown how the skills of the pharmacist can be modeled and adapted to circumstances, in order to alleviate the pressure of physicians, optimize treatment and work collaboratively to address the important challenges of this pandemic and its aftermath.

## 13. Conclusions

During the last decades, the role of the pharmacist has shifted from being products-based and patient-facing to being services-based and patient-centered. Pharmacies have transitioned from being compounding centers devoted to the manipulation of *materia medica* to pharmaceutical centers, clinical pharmacies and fully integrated “medical-pharmaceutical networks”, providing a significant range of non-prescribing services. Moreover, roles, duties and responsibilities of pharmacists have paralleled such historical changes and have known a gradual extension, incorporating new skills and reflecting new societal demands and challenges.

The COVID-19 outbreak has unearthed new opportunities for pharmacists and has propelled new changes: community and hospital pharmacists have, indeed, stepped in new roles and have played a key role during the COVID-19 pandemic, mitigating its devastating outcomes and suggesting that a fully integrated, inter-sectoral and inter-professional collaboration is necessary to face crises and public health emergencies.

Preliminary, emerging evidence seems to suggest that, probably, a new era in the history of pharmacies (“the post-COVID-19 post-pharmaceutical care era”) has begun, with community pharmacists acquiring more professional standing, being authentic heroes and integral members of the frontline health workforce. In other words, the health emergency has made it clear that guaranteeing support and care for all patients requires the commitment and presence on the scene of a plurality of skills and health figures—which, with respect to the various required skills, can achieve an evolving health scheme. Finally, policy- and decision-makers should consider the increased SOP of community pharmacists and regulate it by means of juridical provisions.

## Figures and Tables

**Table 1:** Roles, responsibilities, and duties of hospital and community pharmacists as evolved throughout the story.

Possible Roles, Responsibilities and Duties of Pharmacists	References
Pharmacists as experts in drugs and medicines (products-based, patient-facing role)	[10,11]
Healthcare provider and caregiver (client-/patient-centered; services-based role)	[10,11]
Educator and counselor	[15]
Mentor	[15]
Manager	[10,11]
Leader, business/services developer	[10,11]
Researcher/scholar	[15]
Health-related actor and stakeholder	[10,11]

**Table 2:** Roles of hospital and community pharmacists during the COVID-19 pandemic.

**Roles of Pharmacists During the COVID-19 Crisis**
Ensure a stable supply, storage, distribution and prescription of drugs, guaranteeing continuity-of-care
Ensuring medicines refilling and renewal, especially to vulnerable groups and chronically ill patients
Ensure a stable supply of alcohol-based hand sanitizers and personal protective equipment (PPE), such as gloves and face and surgical masks
Enhance and improve pharmacy services, also from remote (tele-pharmacy and tele-health consulting)
Serve as an information hub, provide clients with updated, high-quality information about COVID-19
Educate clients about personal and environmental hygiene and good safety practices
Counteract and combat misinformation concerning COVID-19
Risk assessment, screening, triage, detection, reporting, referral and management of potential COVID-19 cases
Create a pharmacist network for sharing experiences
Take part in drafting guidance, checklist, and guideline documents
Take part in COVID-19 related risk assessment programs
Take part into the design and implementation of clinical trials
Together with other relevant, health-related stakeholders and actors, implement public health programs
Achieve Universal Health Coverage and counteract health inequity and guarantee access to essential healthcare services
**Roles of Pharmacists in the Post-COVID-19 World**
Together with other relevant, health-related stakeholders and actors, follow the easing and lifting of lockdown induced strictures
Promote and enhance public and global health
Be an instrumental part of immunization campaigns

Abbreviations: COVID-19 (coronavirus disease 2019).

## References

[1] Al-Qahtani A.A. (2020). Severe Acute Respiratory Syndrome Coronavirus 2 (SARS-CoV-2): Emergence, History, Basic and Clinical Aspects. Saudi J. Biol. Sci..

[2] Balla M., Merugu G.P., Patel M., Koduri N.M., Gayam V., Adapa S., Naramala S., Konala V.M. (2020). COVID-19, Modern Pandemic: A Systematic Review From Front-Line Health Care Providers’ Perspective. J. Clin. Med. Res..

[3] Nussbaumer-Streit B., Mayr V., Dobrescu A.I., Chapman A., Persad E., Klerings I., Wagner G., Siebert U., Christof C., Zachariah C. (2020). Quarantine alone or in combination with other public health measures to control COVID-19: A rapid review. Cochrane Database Syst. Rev..

[4] Kennedy M.J. (2018). Personalized medicines—Are pharmacists ready for the challenge?. Integr. Pharm. Res. Pract..

[5] Youssef E., Wright D. (2020). Pharmacogenomic testing and its future in community pharmacy. Pharm. J..

[6] Skolaut M.W. (1980). Moving toward the 21st century. Am. J. Health Pharm..

[7] Traulsen J.M., Druedahl L.C. (2018). Shifting perspectives—Planning for the future of the pharmacy profession taking current labor market trends into consideration. Res. Soc. Adm. Pharm..

[8] John C. (2018). The changing role of the pharmacist in the 21st century. Pharm. J..

[9] Urick B.Y., Meggs E.V. (2019). Towards a Greater Professional Standing: Evolution of Pharmacy Practice and Education, 1920-2020. Pharmacy.

[10] Toklu H.Z., Hussain A. (2013). The changing face of pharmacy practice and the need for a new model of pharmacy education. J. Young Pharm..

[11] Carter B.L. (2016). Evolution of Clinical Pharmacy in the US and Future Directions for Patient Care. Drugs Aging.

[12] Behzadifar M., Ghanbari M.K., Bakhtiari A., Behzadifar M., Bragazzi N.L. (2020). Ensuring adequate health financing to prevent and control the COVID-19 in Iran. Int. J. Equity Health.

[13] Klepser M.E., Klepser D.G., Dering-Anderson A., Morse J.A., Smith J.K., Klepser S.A. (2016). Effectiveness of a pharmacist-physician collaborative program to manage influenza-like illness. J. Am. Pharm. Assoc..

[14] Chin T.W., Chant C., Tanzini R. (2004). Severe acute respiratory syndrome (SARS): The pharmacist’s role. Pharmacotherapy.

[15] Merks P., Jakubowska M., Drelich E., Świeczkowski D., Bogusz J., Bilmin K., Sola K.F., May A., Majchrowska A., Koziol M. (2020). The legal extension of the role of pharmacists in light of the COVID-19 global pandemic. Res. Soc. Adm. Pharm..

[16] Ung C.O.L. (2020). Community pharmacist in public health emergencies: Quick to action against the coronavirus 2019-nCoV outbreak. Res. Soc. Adm. Pharm..

[17] Tan S.L., Zhang B.K., Xu P. (2020). Chinese pharmacists’ rapid response to the COVID-19 outbreak. Am. J. Health Syst. Pharm..

[18] McConachie S.M., Martirosov D., Wang B., Desai N., Jarjosa S., Hsaiky L. (2020). Surviving the surge: Evaluation of early impact of COVID-19 on inpatient pharmacy services at a community teaching hospital. Am. J. Health Pharm..

[19] Liu S., Luo P., Tang M., Hu Q., Polidoro J.P., Sun S., Gong Z.-C. (2020). Providing pharmacy services during the coronavirus pandemic. Int. J. Clin. Pharm..

[20] Bragazzi N.L., Dai H., Damiani G., Behzadifar M., Martini M., Wu J. (2020). How Big Data and Artificial Intelligence Can Help Better Manage the COVID-19 Pandemic. Int. J. Environ. Res. Public Health.

[21] Crowson M.G., Schulz K., Tucci D.L. (2016). National Utilization and Forecasting of Ototopical Antibiotics. Medicaid Data Versus “Dr. Google”. Otol. Neurotol..

[22] Simmering J.E., Polgreen L.A., Polgreen P.M. (2014). Web search query volume as a measure of pharmaceutical utilization and changes in prescribing patterns. Res. Soc. Adm. Pharm..

[23] Schuster N.M., Rogers M.A.M., McMahon L.F. (2010). Using search engine query data to track pharmaceutical utilization: A study of statins. Am. J. Manag. Care.

[24] Wang Y., Lipner S.R. (2020). Retrospective analysis of adverse events with topical onychomycosis medications reported to the United States Food and Drug Administration. Arch. Dermatol. Res..

[25] Springer S., Menzel L.M., Zieger M. (2020). Google Trends reveals: Focus of interest in the population is on treatment options rather than theories about COVID-19 animal origin. Brain, Behav. Immun..

[26] Springer S., Menzel L.M., Zieger M. (2020). Google Trends provides a tool to monitor population concerns and information needs during COVID-19 pandemic. Brain Behav. Immun..

[27] Kouzy R., Jaoude J.A., Kraitem A., El Alam M.B., Karam B., Adib E., Zarka J., Traboulsi C., Akl E.W., Baddour K. (2020). Coronavirus Goes Viral: Quantifying the COVID-19 Misinformation Epidemic on Twitter. Cureus.

[28] Rovetta A., Bhagavathula A.S. (2020). COVID-19-Related Web Search Behaviors and Infodemic Attitudes in Italy: Infodemiological Study. JMIR. Public Health Surveill..

[29] Alkhawajah A.M., Eferakeya A.E. (1992). The role of pharmacists in patients’ education on medication. Public Health.

[30] Asfaw D.S., Belachew S.A., Abrha S., Sinnollareddy M., Thomas J., Steadman K.J., Tesfaye W.H. (2020). When fear and misinformation go viral: Pharmacists’ role in deterring medication misinformation during the ‘infodemic’ surrounding COVID-19. Res. Soc. Adm. Pharm..

[31] Schillinger D., Chittamuru D., Ramírez A.S. (2020). From “Infodemics” to Health Promotion: A Novel Framework for the Role of Social Media in Public Health. Am. J. Public Health.

[32] Tangcharoensathien V., Calleja N., Nguyen T., Purnat T., D’Agostino M., Garcia Saiso S., Landry M., Rashidian A., Hamilton C., AbdAllah A. (2020). A Framework for Managing the COVID-19 Infodemic: Methods and Results of an Online, Crowdsourced WHO Technical Consultation. J. Med. Internet Res..

[33] Eysenbach G. (2020). How to Fight an Infodemic: The Four Pillars of Infodemic Management. J. Med Internet Res..

[34] Li H., Zheng S., Liu F., Liu W., Zhao R. (2020). Fighting against COVID-19: Innovative strategies for clinical pharmacists. Res. Soc. Adm. Pharm..

[35] Feng W., Zhang L.N., Li J.Y., Wei T., Peng T.T., Zhang D.X., Guo Z.X., Wang W.S. (2020). Analysis of Special Ehealth Service for Corona Virus Disease 2019 (COVID-19) Pneumonia. Beijing Da Xue Xue Bao Yi Xue Ban.

[36] Yemm K.E., Arnall J.R., Cowgill N.A. (2020). Necessity of Pharmacist-driven non-prescription telehealth consult services in the era of COVID-19. Am. J. Health Syst. Pharm..

[37] Margusino-Framiñán L., Illarro-Uranga A., Lorenzo-Lorenzo K., Monte-Boquet E., Márquez-Saavedra E., Fernández-Bargiela N., Gómez-Gómez D., Lago-Rivero N., Poveda-Andrés J.L., Díaz-Acedo R. (2020). Pharmaceutical care to hospital outpatients during the COVID-19 pandemic. Telepharmacy. Farm. Hosp..

[38] Hoti K., Jakupi A., Hetemi D., Raka D., Hughes J., Desselle S. (2020). Provision of community pharmacy services during COVID-19 pandemic: A cross sectional study of community pharmacists’ experiences with preventative measures and sources of information. Int. J. Clin. Pharm..

[39] Hamza M.S., Badary O.A., Elmazar M.M. (2020). Cross-Sectional Study on Awareness and Knowledge of COVID-19 Among Senior pharmacy Students. J. Community Health.

[40] Karasneh R., Al-Azzam S., Muflih S., Soudah O., Hawamdeh S., Khader Y. (2020). Media’s effect on shaping knowledge, awareness risk perceptions and communication practices of pandemic COVID-19 among pharmacists. Res. Soc. Adm. Pharm..

[41] Saqlain M., Munir M.M., Rehman S.U., Gulzar A., Naz S., Ahmed Z., Tahir A.H., Mashhood M. (2020). Knowledge, attitude, practice and perceived barriers among healthcare professionals regarding COVID-19: A Cross-sectional survey from Pakistan. J. Hosp. Infect..

[42] Bahlol M., Dewey R.S. (2020). Pandemic preparedness of community pharmacies for COVID-19. Res. Soc. Adm. Pharm..

[43] Elbeddini A., Prabaharan T., Almasalkhi S., Tran C. (2020). Pharmacists and COVID-19. J. Pharm. Policy Pract..

[44] Alderman C. (2020). Pharmacy Services and the Novel Coronavirus. Sr. Care Pharm..

[45] Luisetto M. (2016). Clinical pharmacist active role in registrative clinical trials. AJPP.

[46] Gross A.E., MacDougall C. (2020). Roles of the clinical pharmacist during the COVID-19 pandemic. ACCP.

[47] Tsang J.L.Y., Binnie A., Farjou G., Fleming D., Khalid M., Duan E. (2020). Participation of more community hospitals in randomized trials of treatments for COVID-19 is needed. CMAJ.

[48] Castro-Balado A., Varela-Rey I., Bandín-Vilar E.J., Busto-Iglesias M., García-Quintanilla L., Mondelo-García C., Fernández-Ferreiro A. (2020). Clinical research in hospital pharmacy during the fight against COVID-19. Farm. Hosp..

[49] Martin R., Löchel H.F., Welzel M., Hattab G., Hauschild A.-C., Heider D. (2020). CORDITE: The Curated CORona Drug InTERactions Database for SARS-CoV-2. iScience.

[50] Sakeena M.H.F., Bennett A.A., McLachlan A.J. (2019). The Need to Strengthen the Role of the Pharmacist in Sri Lanka: Perspectives. Pharmacy.

[51] Burke R. (2020). Embracing the Evolution of Pharmacy Practice by Empowering Pharmacy Technicians. Pharmacy.

[52] Hitch W.J., Ulrich I.P., Warren A.C., Stick D., Leyonmark D., Farrar M. (2019). Evolution of Interdisciplinary Transition of Care Services in a Primary Care Organization. Pharmacy.

[53] Nguy J., Hitchen S.A., Hort A.L., Huynh C., Rawlins M.D.M. (2020). The role of a Coronavirus disease 2019 pharmacist: An Australian perspective. Int. J. Clin. Pharm..

[54] Dawoud D. (2020). Emerging from the other end: Key measures for a successful COVID-19 lockdown exit strategy and the potential contribution of pharmacists. Res. Soc. Adm. Pharm..

